# Supplementary material for: Evaluating the efficacy of serological testing of clinical specimens collected from patients with suspected brucellosis
Source: PLoS Negl Trop Dis. 2023 Feb 21;17(2):e0011131. doi: 10.1371/journal.pntd.0011131 (PMC9942959; doi:10.1371/journal.pntd.0011131)
Supplement: S3 Material — (DOCX) [file pntd.0011131.s006.docx]

**Constructing the multivariate binary logistic regression model**

We established a multivariate binary logistic regression model with SAT, ELISA IgM, ELISA IgG, and Brucellacapt as independent variables, with or without brucellosis as the dependent variable.

We transformed the categorical variable of Brucellacapt into a rank variable based on titer, with negative assignments of 0, 1:160 positive (1), 1:320 positive (2), 1:640 positive (3), and 1:1280 positive or higher (4). Similarly, we converted categorical variables for the SAT to grade variables based on titer, with negative results assigned as 0, 1:50 positive (1), 1: 100 positive (2), 1: 200 positive (3), and 1: 400 positive or higher (4). Then, we checked whether there was multicollinearity among the independent variables. We used tolerance and variance expansion factors to diagnose multicollinearity among independent variables. Among the included independent variables, the tolerances were much larger than 0.1, and the variance inflation factors were less than 10, so there was no multicollinearity among variables.

Forward stepwise (likelihood ratio) binary logistic regression(1-3) was used to find significant variables and probability of disease prediction. At last, ELISA IgG and Brucellacapt were screened into the model. The area under the receiver operator characteristic curve (AUROC) for distinguishing between brucellosis and other disease were 0.989 (95% confidence interval [CI] 0.969-1.000). The difference in AUC between pairs of ROC curves was evaluated with DeLong’s test. We found that the AUC (Supplementary Figure 1 and Supplementary Table 1) of the model in distinguishing brucellosis from other disease was significantly higher compared with the AUCs of SAT, ELISA IgM, ELISA IgG, and Brucellacapt (P＜0.0001 SAT, P＜0.001 ELISA IgM, P=0.017 ELISA IgG, P=0.005 Brucellacapt). The modeling analysis was carried out using SPSS 24.0 (IBM Corp., Somers, NY, USA) statistical software.

**Determination of the optimal cutoff value for Brucellacapt**

To obtain greater efficiency, we adjusted the cutoff value according to the ROC curve. We transformed the categorical variable of Brucellacapt into a rank variable based on titer, with negative assignments of 0, 1:160 positive (1), 1:320 positive (2), 1:640 positive (3), and 1:1280 positive or higher (4). The AUROC (Supplementary Figure 1) for Brucellacapt was 0.942 (95% CI 0.901-0.983), and the cutoff value based on the best Youden index was ≥ 1:160, with a sensitivity of 88.37% (95% CI 79.90, 93.56) and a specificity of 100% (95% CI 94.25-100).


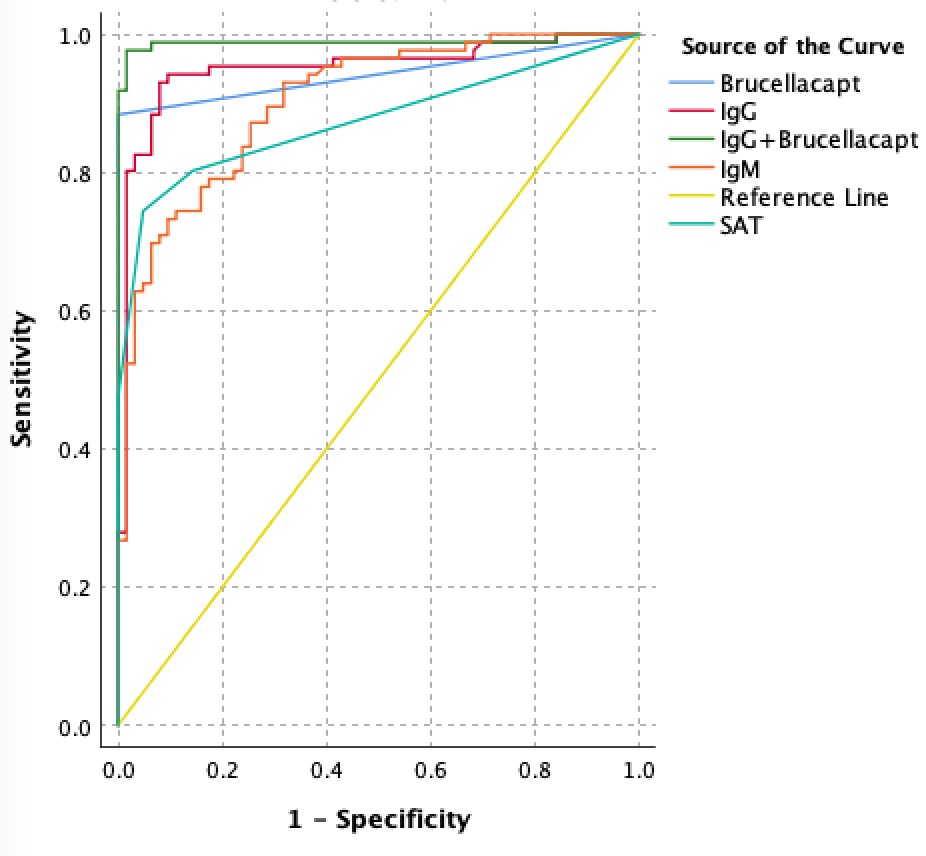


**Supplementary Figure** **1** Receiver operating characteristic curves for each comparison or combination of SAT, ELISA IgM, ELISA IgG, and Brucellacapt.

**Supplementary Table 1** The area under the receiver operator characteristic curve for each comparison or combination of SAT, ELISA IgM, ELISA IgG, and Brucellacapt

| Variable | AUC | 95% CI |
| --- | --- | --- |
| SAT | 0.875 | 0.818-0.932 |
| IgM | 0.903 | 0.856-0.950 |
| IgG | 0.950 | 0.912-0.988 |
| Brucellacapt | 0.942 | 0.901-0.983 |
| IgG+Brucellacapt | 0.989 | 0.969-1.000 |

Abbreviations: SAT, standard tube agglutination; IgM, IgM antibody detection by ELISA; IgG, IgG antibody detection by ELISA; AUC, area under the receiver operator characteristic curve; 95% CI, 95% confidence interval.

**References**

1. Varpula M, Tallgren M, Saukkonen K, Voipio-Pulkki LM, Pettila V. Hemodynamic variables related to outcome in septic shock. Intensive Care Med. 2005;31(8):1066-71.

2. Gupta PK, Prabhakar S, Sharma S, Anand A. A predictive model for amyotrophic lateral sclerosis (ALS) diagnosis. J Neurol Sci. 2012;312(1-2):68-72.

3. Li CK, Xu Z, Ho J, Lakhani I, Liu YZ, Bazoukis G, et al. Association of NPAC score with survival after acute myocardial infarction. Atherosclerosis. 2020;301:30-6.
