# Supplementary material for: Evaluating the efficacy of serological testing of clinical specimens collected from patients with suspected brucellosis
Source: PLoS Negl Trop Dis. 2023 Feb 21;17(2):e0011131. doi: 10.1371/journal.pntd.0011131 (PMC9942959; doi:10.1371/journal.pntd.0011131)
Supplement: Attachment — Submitted filename: Responses to Reviewer Comments.docx [file pntd.0011131.s007.docx]

1. Response to comment：The supplementary table is not very useful in its present form.  First, the serological results should be given in the table for all the patients, second, the patients where a Brucella strain was isolated should be identified. Finally, rather than having the patients listed by number, please list them by duration of illness at diagnosis.

**Response:** We appreciate this good suggestion. According to your suggestion, we added the corresponding information to Supplementary Material 1.

2. Response to comment：L123.  you read the Absorbance A450, not optical density,  Same for line 127.

**Response:** We made the correction following the reviewer’s suggestion; see lines 127, 131 and 132.
3. Response to comment：There were 31 positive blood cultures and one synovial fluid. Does this represent 32 patients, or were there multiple positive samples form patients?

**Response:** We apologize for the confusion. In this study, these 32 positive samples were from 32 patients. We have made this clear in our revised manuscript; see line 193. In addition, You can also find patients with the Brucella isolated in Supplement Material 2.
4. Response to comment：L156, what is a case of 'nonbrucellosis;? This is not a disease, please reword. As you use these patients as a control group, please describe which infections these patients had. this is important to rule out the possibility of cross reactivity.

**Response:** We completely agree with the editor. We have carefully modified these in our revised manuscript; see lines 164-166,168-169,173,184-185, and Table 1.

5. Response to comment：I am in full agreement with reviewer 1, that it is ESSENTIAL to include an age matched healthy control group.  This is also essential when describing the changes in blood biochemistry.

**Response:** We completely agree with the editor and reviewers. A group of 50 healthy people were enrolled in this study, and the antibody levels were also tested, see lines 100-102. Only one individual was positive for IgM, but negative for both SAT and IgG; no individuals had Brucellacapt titers of ≥ 1:160, see lines 204-205, and 232-233. Detailed serological results for the healthy control group were provided in Supplementary Material 2.

6. Response to comment：L 223 -6 and 299-9  The manufacturers instructions say that the limit of positivity with BrucellaCapt is 1/160.  This is obviously based on extensive testing, what is the rational to alter the criteria to 1/320? It is evident that this will decrease the sensitivity and NPV.

**Response:** Due to regional differences in the prevalence of antibodies to Brucella, it is important to establish a “normal range” for the local population. Therefore, the manufacturer recommends that one or more cutoff values should be selected for diagnosis based on the local brucellosis prevalence.

In a number of related studies (PMID: 19625085; PMID: 18448622; PMID: 31960174, etc.), researchers recommended a Brucellacapt titer ≥ 1/320 as the diagnostic threshold, which has high sensitivity and specificity. To obtain greater efficiency, we adjusted the cutoff value according to the ROC curve. We transformed the categorical variable of Brucellacapt into a rank variable based on titer; see the Supplementary Material 3 for details. The AUROC for Brucellacapt was 0.942 (95% CI 0.901-0.983), and the cutoff value based on the best Youden index was ≥1:160. A titer ≥1/160 in Brucellacapt indicates the existence of brucellosis with a high degree of probability, and titers lower than these allow us to eliminate the possibility of brucellosis in the majority of cases.

7. Response to comment：On the same line, you define 1/100 as positive with the SAT.  I know that this is the titer defined by Chinese Law, but it is different from the recommendations in other countries (1/80; 120IU).  How does difference effect your analysis?  Please also indicate which positive serum was used and whether it was calibrated?

**Response:** As the editor mentioned, there is a lack of consensus about the diagnostic endpoint titer of SAT (1): the WHO and US CDC have set the minimum antibody endpoint titer at 1:160, but the minimum antibody endpoint titer in China is 1:100.

We searched the literature database, but there were no systematic studies comparing the different end-point titers of SAT in China and other countries. In addition, these assays are not yet available in China. Therefore, we were unable to evaluate the impact of different diagnostic criteria on the analysis results.

However, as the editor mentioned, ≥1/100 as the diagnostic endpoint titer is obviously based on extensive testing and has been widely used for the clinical diagnosis and studies of human brucellosis in China (2-4).

The positive control and antigen of the SAT assays were provided by the China CDC, and the methods were subject to the Diagnostic Criteria for Brucellosis (WS 269-2019).

References

1.	Jiang H, Feng L, Lu J. Updated Guidelines for the Diagnosis of Human Brucellosis - China, 2019. China CDC Wkly. 2020;2(26):487-9.

2.	Zhang S, Hu J, An S, Li M, Li F, Zhang P, et al. Prevalence and relevant factors of positive RF in brucellosis patients with arthralgia. PLoS Negl Trop Dis. 2021;15(9):e0009749.

3.	Wang H, Liu H, Zhang Q, Lu X, Li D, Zhang H, et al. Natural History of and Dynamic Changes in Clinical Manifestation, Serology, and Treatment of Brucellosis, China. Emerg Infect Dis. 2022;28(7):1460-5.

4.	Tuohutaerbieke M, Li X, Yin Y, Chen W, Wu D, Mao Z, et al. The Characteristics, Prevalence, and Risk Factors of Drug-Induced Liver Injury Among Brucellosis Inpatients in Xinjiang, China. Front Pharmacol. 2021;12:657805.

8. Response to comment：L301-2  You comment that some chronic patients were negative with BrucellaCapt.  Please support this by giving the full serological and culture results of these patients in a table rather than leaving the reader to find it in the supplemental table

**Response:** Following the reviewer’s suggestion, we have included detailed information on the serological and culture results of all patients in Supplementary Material 1.

9. Response to comment：Reviewer #1: The methods are clear although the criterion used to select the Brucellosis infection samples should be clarified and a true group of negative samples tested (see comments to authors).

**Response:** The criteria we made were based on those of the US CDC. The diagnosis of brucellosis was based on the proper clinical context, including history, clinical presentation and laboratory studies as well as at least one of the following results being positive: bacterial culture or fourfold or greater increase in SAT titer; see lines 31 and 108. We used paired serum for serological diagnosis. The first serum sample was collected from the patient at the time of recruitment; the second serum sample was drawn 2-4 weeks later to check for an increase in antibodies. We have carefully modified our revised manuscript; see lines 99-100.

In addition, a group of 50 healthy people were enrolled in this study, and the antibody levels were also tested. Only one individual was positive for IgM, but negative for both SAT and IgG; no individuals had Brucellacapt titers of ≥ 1:160, see lines 204-205, and 232-233. Considering that in actual clinical practice, all patients who need differential diagnosis are symptomatic, we have not described these results in detail in this manuscript. All serological results for the healthy control group were provided in Supplementary Material 2.

10. Response to comment：Reviewer #2: McNamar’s test - please check the spelling
Was the study multicentric? or conducted in a single hospital? The information is not clear
How the specificity, sensitivity, and positive (PPVs) and negative predictive values (NPVs) were calculated? Using a frequentist approach I presume. It should be clearly explained in M&M and the authors should provide a reference for the calculation.
Were confidence interval calculated? How?

**Response:** We truly appreciate the reviewer’s careful reading of our manuscript. We have checked and corrected this error, see line 154.This was a single-center study conducted in Qilu Hospital of Shandong University, see lines 95-96. The sensitivity, specificity, PPV and NPV were obtained with OpenEpi version 3.0 (http://www.openepi.com/). The 95% confidence intervals were calculated using Wilson’s method. Following the reviewer’s suggestions, we have now included detailed information in the M&M, see lines 149-153.

11. Response to comment：Reviewer #1: The results are simple and clear but they should be reinterpreted after it is clarified how the positive samples were selected and, if necessary, modified accordingly. The same when a true negative group of samples are evaluated.

**Response:** The criteria we made were based on those of the US CDC. The diagnosis of brucellosis was based on the proper clinical context, including history, clinical presentation and laboratory studies as well as at least one of the following results being positive: bacterial culture or fourfold or greater increase in SAT titer; see lines 31 and 108. We used paired serum for serological diagnosis. The first serum sample was collected from the patient at the time of recruitment; the second serum sample was drawn 2-4 weeks later to check for an increase in antibodies. We have carefully modified our revised manuscript; see lines 99-100. Considering that in actual clinical practice, all patients who need differential diagnosis are symptomatic, so we still have taken the patients without brucellosis as the control group. These modifications have no impact on the results, and no changes are required for subsequent analysis.

12. Response to comment：Reviewer #2: The other criteria used for the classification of cases without isolation should be clearly mentioned. How many patients were PCR positive among the cases for example?

**Response:** The criteria we made were based on those of the US CDC. We used paired serum for serological diagnosis. The first serum sample was collected from the patient at the time of recruitment; the second serum sample was drawn 2-4 weeks later to check for a rise in antibodies. In actual clinical practice, we found that the diagnosis of brucellosis cannot be made with a single serum sample. However, the dynamic increase in antibodies in paired serum often lags behind in the diagnosis of brucellosis. Therefore, we would like to screen out other serological methods or combinations that are superior to SAT for the diagnosis of brucellosis when a person is acutely ill.

PCR technology has not been included in the diagnostic criteria of brucellosis in China and other countries. PCR detection kits for brucellosis have not been approved, and commercial kits have not been widely used because of differences in diagnostic performance. The sensitivity and accuracy of PCR-based methods are dependent on the DNA extraction method and the quality of the extracted genomic DNA. Another problem widely associated with the use of PCR was the possibility of contamination during the processing of samples, which has the potential to lead to false positives. In addition, the sensitivity of PCR-based methods can be reduced in chronic brucellosis.

Culture is the gold standard method for Brucella detection, but the positive rate of culture is very low and is influenced by factors such as the course of disease, antibiotics and sample collection. Relying solely on culture would miss a large number of patients with chronic disease.

Serological tests are imperfect but indispensable.

13. Response to comment：The univariate analysis of the variables possibly associated with brucellosis is poorly suited to the inference of risk factors as it does not consider confounding factors, collinearity, etc. A multivariate model would be more suitable and informative.

**Response:** According to the reviewer’s suggestions, we have constructed a multivariate model; see the Supplementary Material 3 for details. Four independent variables, namely, SAT, ELISA IgM, ELISA IgG and Brucellacapt, were included in the regression model. We determined the AUC of the model with each of these indicators and found that the diagnostic efficiency of the model was significantly better than that of a single indicator. However, there was no significant difference between this model and the combined IgG+Brucellacapt in diagnostic efficacy.
14. Response to comment：How the results of different tests were combined? Considering a parallel testing? In this case how the final sensitivity and specificity were calculated? Did the authors considered that the results of the test are not indenpedent? Was the covariance between the tests estimated and considered in the calculation of combined Se and Sp?

**Response:** Serological methods are indispensable for the diagnosis of brucellosis, but a single serological test may not meet the needs of clinical diagnosis. We used three different methods to detect the same serum sample to improve the sensitivity or specificity of the diagnosis. As long as any test result was positive, it was considered positive, and only when the results of all tests were negative was it considered negative (parallel tests).

This situation has an effect on the sensitivity, specificity and ultimately the applicability of the different methods. The various test results are independent from each other, and the main purpose of performing parallel tests in this study is to improve the sensitivity or specificity of diagnosis as much as possible.

For the covariance between the tests the reviewer mentioned, we found that the SAT and Brucellacapt data in this study did not meet the requirements of covariance analysis; that is, covariate and dependent variables were continuous data. In addition, binomial logistic regression analysis showed that there was no multicollinearity between independent variables, see the Supplementary Material 3.

15. Response to comment：Figure would be of great help to understand the data

**Response:** We completely agree with the editor and reviewers. We made changes in our manuscript content and added graphics to better represent the results of our study.

see the Supplementary Material 3.

16. Response to comment：Reviewer #1: As with the results conclusions should be reevaluated once it is clarified how the positive samples were selected and, if necessary, modified accordingly. The same when a true negative group of samples are evaluated.

**Response:** The criteria we made were based on those of the US CDC. The diagnosis of brucellosis was based on the proper clinical context, including history, clinical presentation and laboratory studies as well as at least one of the following results being positive: bacterial culture or fourfold or greater increase in SAT titer; see lines 31 and 108. We used paired serum for serological diagnosis. The first serum sample was collected from the patient at the time of recruitment; the second serum sample was drawn 2-4 weeks later to check for an increase in antibodies. We have carefully modified our revised manuscript; see lines 99-100. Considering that in actual clinical practice, all patients who need differential diagnosis are symptomatic, so we still have taken the patients without brucellosis as the control group. These modifications have no impact on the results, and no changes are required for subsequent analysis.

17. Response to comment：Reviewer #2: The analysis should be reviewed considering that the tests are not independent (they are all measuring anti-brucella antibodies)

**Response:** The criteria we made were based on those of the US CDC. We used paired serum for serological diagnosis. The first serum sample was collected from the patient at the time of recruitment; the second serum sample was drawn 2-4 weeks later to check for an increase in antibodies.

In actual clinical practice, we found that the diagnosis of brucellosis cannot be made with a single serum sample. However, the dynamic increase in antibodies in paired serum often lags behind in the diagnosis of brucellosis. Therefore, we hope to further determine other serological methods or combinations that are superior to SAT in this study.

18. Response to comment：Reviewer #1: 1- What it is not clear is what criteria was used to diagnose brucellosis on the 149 patients. They state that a patient was diagnosed with brucellosis if it was blood culture positive or had a positive SAT. With this criterion 86 patients out of the 149 were included as positives. 32 were blood culture positive and 26 of these had SAT positive implying that 5 were negative which would mean that, out of the 86 considered as Brucellosis positive, 81 should be SAT positive. This is not what Table 2 shows. There it is stated that 64 of the 86 samples were SAT positive. This is all very confusing, if the criteria to diagnose brucellosis was SAT positive how is it possible that the sensitivity of the method is after this selection was only 74%? The correct way to evaluate this is to take one test as the gold standard and then compare the other ones against this group. As it is described, the analysis is very confusing.

**Response:** Some of our descriptions we made were more ambiguous than intended.

First, the criteria in our study were based on those of the US CDC (<https://www.cdc.gov/brucellosis/clinicians/serology.html>). The diagnosis of brucellosis was based on the proper clinical context, including history, clinical presentation and laboratory studies, as well as at least one of the following results being positive: bacterial culture or a fourfold or greater increase in SAT titer. We used paired serum for serological diagnosis. The first serum sample was collected from the patient at the time of recruitment; the second serum sample was drawn 2-4 weeks later to check for an increase in antibodies. We used the first sample to compare the diagnostic efficacy of different serological testing. Some patients initially tested negative for SAT and had more than 4-fold increases in antibody titers 2 to 4 weeks later. For this reason, some patients with brucellosis had negative SAT serological results at the time of registration.

The diagnosis of brucellosis is often delayed due to the dynamic increase in antibodies in paired sera. Therefore, we would like to screen out other serological methods or combinations that are superior to SAT for the diagnosis of brucellosis when a person is acutely ill.

Culture is the gold standard method for Brucella detection, but the positive rate of culture is very low and is influenced by factors such as the course of disease, antibiotics and sample collection. Relying solely on culture would miss a large number of patients with chronic disease. Serological tests are imperfect but indispensable.

19. Response to comment：2- The negative samples also have problems as all these samples come from potential infections (symptomatic). A true negative control group should be sera from healthy individuals.

**Response:** We understand the concerns of the reviewer. A group of 50 healthy people were enrolled in this study, and the antibody levels were also tested. Only one individual was positive for IgM, but negative for both SAT and IgG; no individuals had Brucellacapt titers of ≥ 1:160, see lines 204-205, and 232-233. All serological results for the healthy control group were provided in Supplementary Material 2.

Considering that in actual clinical practice, all patients who need differential diagnosis are symptomatic, so we still have taken the patients without brucellosis as the control group.

20. Response to comment：Reviewer #2: it is an interesting article as it uses prospective sampling to assess the diagnostic sensitivity and specificity of serological tests for human brucellosis. In this sense, as it uses the same tests for definition of cases and non-cases, it has greater reilability of estimation than studies conducted in the case control format. However, the statistical approach used for the analysis is unclear and seems inadequate.

**Response:** Thank you for the suggestion. Several statements that we made were more ambiguous than intended. We have further revised and added some statistical method information in the “Materials and Methods” section and the Supplementary Materials in the hope of making it clearer; see lines 149-153 and Supplementary Material 3.
