# Supplementary material for: Evaluating the efficacy of serological testing of clinical specimens collected from patients with suspected brucellosis
Source: PLoS Negl Trop Dis. 2023 Feb 21;17(2):e0011131. doi: 10.1371/journal.pntd.0011131 (PMC9942959; doi:10.1371/journal.pntd.0011131)
Supplement: Attachment — Submitted filename: Responses to Reviewer Comments.docx [file pntd.0011131.s008.docx]

1.Response to comment：Reviewer #2: the "multivariate" statistical model used was not properly described, neither a description of the model nor of the references used. In addition, in the combinations of tests evaluated, the tests were considered independent to calculate Se and Sp, which is not true (all are basically measuring the same thing), the probability of the occurrence of one result is associated with the probability of another test. Thus, it would be necessary to evaluate the covariance between them and consider the value in the simulations of use in parallel.

**Response:** Thank you for the suggestion. According to the opinions of the first revision and the second revision, we now give an appropriate explanation to your question.

Our research is a diagnostic study, and the original intention is to observe the diagnostic performance of single/combined tests for brucellosis, and not to identify independent predictors of brucellosis or establish disease diagnosis models through multivariate analysis. In this study, the evaluation indicators of diagnostic performance include sensitivity, specificity, positive predictive value and negative predictive value. Parallel tests were used in our study to reduce the rate of missed diagnosis of brucellosis, and to facilitate early treatment (PMID:12695154，PMID:27800636). Following the reviewer’s suggestion, the multivariate model was also reconstructed, and described in detail in the supplementary material, and additional references were added, as described in Supplementary Material 3.

SAT tests all agglutinating antibodies (including IgM, IgA and IgG) against S-LPS antigen of *Brucella*. The ELISA kits were used for detecting IgM and IgG antibodies specific to *Brucella* LPS. Brucellacapt is an assay for the detection of total anti-*Brucella* antibodies (non-agglutinating and agglutinating antibodies). The three tests being based on quite different techniques (PMID:17118583; PMID:17590540, PMID:29290297). Knowing SAT test result, will not change our belief of a positive (or negative) test result of the ELISA and Brucellacapt. The assumption that the three test are conditionally independent is justified by the three tests since they are based on different techniques.

2.Response to comment：Results Reviewer #2: biased based on the statistical approach used

**Response:** Thank you for the suggestion. As mentioned in response to #1, we now mentioned this limitation of analysis in the discussion. We believe that our revisions have significantly improved our manuscript.

3.Response to comment：Conclusions

-Are the conclusions supported by the data presented?

-Are the limitations of analysis clearly described?

-Do the authors discuss how these data can be helpful to advance our understanding of the topic under study?

-Is public health relevance addressed?

Reviewer #2: either

**Response:** Thank you for the suggestion. We have revised the conclusion of our manuscript. The limitations of analysis were also added in the discussion. We believe that our revisions have significantly improved our manuscript.
